# Previous Infection with SARS-CoV-2 Correlates with Increased Protective Humoral Responses after a Single Dose of an Inactivated COVID-19 Vaccine

**DOI:** 10.3390/v14030510

**Published:** 2022-03-02

**Authors:** Flávia F. Bagno, Luis A. F. Andrade, Sarah A. R. Sérgio, Pierina L. Parise, Daniel A. Toledo-Teixeira, Ricardo T. Gazzinelli, Ana P. S. M. Fernandes, Santuza M. R. Teixeira, Fabiana Granja, José L. Proença-Módena, Flavio G. da Fonseca

**Affiliations:** 1 Centro de Tecnologia de Vacinas, Universidade Federal de Minas Gerais (UFMG), Belo Horizonte 31270-901, Brazil; flavia.bagno@gmail.com (F.F.B.); luisadanflores@gmail.com (L.A.F.A.); sarsergio36@gmail.com (S.A.R.S.); ricardo.gazzinelli@fiocruz.br (R.T.G.); apfernandes.ufmg@gmail.com (A.P.S.M.F.); santuzat@icb.ufmg.br (S.M.R.T.); 2 Laboratory of Emerging Viruses, Department of Genetics, Evolution, Microbiology and Immunology, University of Campinas, Campinas 13083-862, Brazil; pierinalp@gmail.com (P.L.P.); teixeiradatt@gmail.com (D.A.T.-T.); fabi.granja@yahoo.com.br (F.G.); jlmodena@unicamp.br (J.L.P.-M.); 3 Fundação Oswaldo Cruz-Fiocruz, Centro de Pesquisas René Rachou, Belo Horizonte 30190-002, Brazil; 4 Biodiversity Research Center, Federal University of Roraima, Boa Vista 72000-000, Brazil; 5 Experimental Medicine Research Cluster, State University of Campinas, Campinas 13083-862, Brazil

**Keywords:** COVID-19, CoronaVac, antibodies, PRNT, ELISA

## Abstract

Previous studies have indicated that antibody responses can be robustly induced after the vaccination in individuals previously infected by SARS-CoV-2. To evaluate anti-SARS-CoV-2 humoral responses in vaccinated individuals with or without a previous history of COVID-19, we compared levels of anti-SARS-CoV-2 antibodies in the sera from 21 vaccinees, including COVID-19-recovered or -naïve individuals in different times, before and after immunization with an inactivated COVID-19 vaccine. Anti-SARS-CoV-2-specific antibodies elicited after COVID-19 and/or immunization with an inactivated vaccine were measured by ELISA and Plaque Reduction Neutralizing assays. Antibody kinetics were consistently different between the two vaccine doses for naïve individuals, contrasting with the SARS-CoV-2-recovered subjects in which we observed no additional increase in antibody levels following the second dose. Sera from SARS-CoV2-naïve individuals had no detectable neutralizing activity against lineage B.1 SARS-CoV-2 or Gamma variant five months after the second vaccine dose. Contrarily, SARS-CoV-2-recovered subjects retained considerable neutralizing activity against both viruses. We conclude that a single inactivated SARS-CoV-2 vaccine dose may be sufficient to induce protective antibody responses in individuals with previous history of SARS-CoV-2 infection.

## 1. Introduction

The COVID-19 vaccine rollout has been decisive for curbing the SARS-CoV-2 pandemic. The impact of vaccination is undeniable, and severe cases of COVID-19 became concentrated in populations that have not been completely immunized [1]. Nonetheless, vaccine rollout has not been equal, and underdeveloped countries struggle to increase their vaccine coverage [2]. The vaccination campaigns make use of vaccines with different characteristics. Two mRNA vaccines have been used. The mRNA BNT162b2/Pfizer and mRNA-1273/Moderna vaccines are based on messenger RNA, or mRNA, -targeting spike glycoprotein from SARS-CoV-2, responsible for the virus/cell attachment. This strategy indices the production of anti-spike antibodies, some of which are able to neutralize the virus by blocking spike binding to ACE2 receptor on type 2 alveolar cells. [3] Viral vectors have also been extensively used in the vaccine rollout. Non-replicative, recombinant adenoviruses were exploited on vaccines by University of Oxford/AstraZeneca and the Janssen Pharmaceuticals. Both are made up of adenovirus vectors encoding the S protein of SARS-CoV-2. After vaccination, it is expected that the surface spike protein is produced, encouraging the immune system to attack when it encounters the SARS-CoV-2 virus [4].

Due to the enormous vaccine demand by developed countries, leading vaccines, such as Pfizer, Moderna, AstraZeneca, and Jansen, are not reaching poorer nations. To fill this gap, less widely known vaccines have been offered to governments, with many of those produced in China [5]. The inactivated whole-virus vaccine CoronaVac (Sinovac Life Science Co., Beijing, China), administrated in two doses with intervals of 2–4 weeks between them, showed an overall efficacy of a little over 50% to prevent symptomatic infection and 84% against COVID-19-related hospitalizations [6] CoronaVac was approved for emergency use in many countries, and most started vaccination with priority given to health care workers, people with comorbidities, and the elderly. In a global context, more than 230 million SARS-CoV-2 cases were reported since the beginning of the pandemic [7], and many of the recovered individuals are being vaccinated. Studies done with vaccinees that received viral vectors or mRNA-based vaccines indicate that antibody responses can be robustly boosted after vaccination of individuals previously infected by SARS-CoV-2 [8,9,10,11].

Therefore, in a context that includes vaccine shortage, mainly in undeveloped or developing countries, would it be necessary to immunize previously infected people with two doses to induce high levels of anti-SARS-CoV2 antibodies? We conducted an exploratory longitudinal study on a small cohort that included SARS-CoV2-naïve individuals and previously infected subjects that had mild COVID-19. All participants received the CoronaVac vaccine. We evaluated the relative amounts of antibodies against the main virus’ antigens and neutralizing antibodies in both groups prior to and after each vaccine dose.

## 2. Materials and Methods

### 2.1. Recruitment and Clinical Sample Collection

We established a longitudinal cohort of SARS-CoV-2 naïve (*n* = 5) and SARS-CoV-2 (mildly symptomatic) -recovered individuals (*n* = 16) who received CoronaVac (Sinovac Life Sciences, Beijing, China) vaccine, of which sera samples were obtained in four time points, including (1) baseline, or before the first vaccine dose, which, for recovered individuals, ranged from 3.6 to 10.9 months after infection (mean = 4.7 months; median = 5 months); (2) two weeks after the prime vaccine dose; (3) three weeks post second vaccine dose; and (4) five months after the second vaccine dose.

### 2.2. Detection of SARS-CoV2-Specific Antibodies

Sera samples were tested for the detection of specific antibodies for Nucleocapsid (N), S1 domain (RBD), and spike (S) protein of SARS-CoV-2 by enzyme-linked immunosorbent assay (ELISA) as previously described [12]. Briefly, ELISA plates (Costar, Ref. 2596) were coated with 4 μg/mL of recombinant proteins and stored overnight at 4 °C. The next day, plates were blocked (PBS with 1% bovine serum albumin) for 2h at room temperature (25 ± 2 °C). For each assay, samples diluted in PBS-T (0.05% Tween-20) were added and incubated for 30 min at 37 °C. After five washes (PBS-T, 1% Tween-20), the conjugate (anti-human IgG, BEEIGG201 Fapon, China, diluted at 1:10,000 in PBS-T) was added, and the plates were further incubated (30 min at 37 °C). After further washing, reactions were revealed using TMB (3,3′,5,5-tetramethylbenzidine, Scienco, Lages, Brazil) for 30 min, and H2SO4 (0.5 M) was added to stop the reaction. Plates were analyzed in a Microplate Reader at an optical density (OD) of 450 nm.

A cut-off value was determined by an absorbance value that was three standard deviations greater than the average OD450 of the negative controls (*n* = 40, samples from healthy donors obtained before 2020). To all validation assays, an index (I) for each sample was calculated, dividing the OD450 of each sample by the cut-off value. The results were classified as non-reactive (I < 0.8), borderline (0.8 ≤ I < 1.1), or reactive (I ≥ 1.1).

### 2.3. PRNT

To access the neutralizing antibodies, an in-house plaque reduction neutralization test (PRNT) was done. E6 Vero cells were maintained in Dulbecco’s Modified Eagle Medium (DMEM) supplemented with 10% fetal bovine serum (FBS) and 100 U/mL of penicillin-streptomycin. The assay was performed in a biosafety level 3 facility. Briefly, the assay was performed in duplicates, conducted days apart, using 24-well tissue culture plates (TPP Techno Plastic Products AG, Trasadingen, Switzerland). The sera were inactivated at 56 °C for 30 min. Serial dilutions of each serum sample were incubated with 150 plaque-forming units of SARS-CoV-2 lineage B.1 11 and/or SARS-CoV-2 Gamma variant 11 for 1 h at 37 °C in a final volume of 200 µL. After incubation, the virus-serum mixtures were added into pre-formed Vero E6 monolayers and incubated for 1 h at 37 °C in 5% CO_2_ incubator. Then, the plates were overlaid with 1% carboximetilcelulose (CMC) in DMEM supplemented with 10% fetal bovine serum (FBS) and 100 U/mL of penicillin-streptomycin and incubated for 72 h at 37 °C in 5% CO_2_, at which time the plates were fixed with 8% formaldehyde solution for 1 h and stained with 1% methylene blue (Sigma-Aldrich, St. Louis, MO, USA) for 30 min. PRNT50 was defined as the highest sample dilution that showed 50% reduction in number of plaques formed compared with positive control consisting of the number of plaques in wells inoculated with SARS-CoV-2 alone. This method using SARS-CoV-2 has been validated previously [13,14]. We tested the sera from SARS-CoV-2-recovered individuals at the following timepoints: (1) baseline; (3) three weeks post second dose; and both groups at timepoint (4) five months after the second vaccine dose.

### 2.4. Statistical Analysis

All data were analyzed using GraphPad Prism Software (version 8.0.1). For ELISA analysis, boxplots represent the median with inter-quartile range. Statistics were calculated using the Kruskal–Wallis test (comparisons between time points) or the unpaired Mann–Whitney test (comparisons between naïve and recovered individuals). All tests were performed two-sided with a nominal significance threshold of *p* < 0.05. * Indicates *p* < 0.05, ** indicates *p* < 0.01, *** indicates *p* < 0.001, and **** indicates *p* < 0.0001. For PRNT analysis, the median for each sample was calculated as a mean of two duplicates. Sera dilutions were transformed in Log(X), and a nonlinear regression (curve fit) was made. Statistical significance between groups medians was determined by *t*-test.

## 3. Results

**Figure 1:**
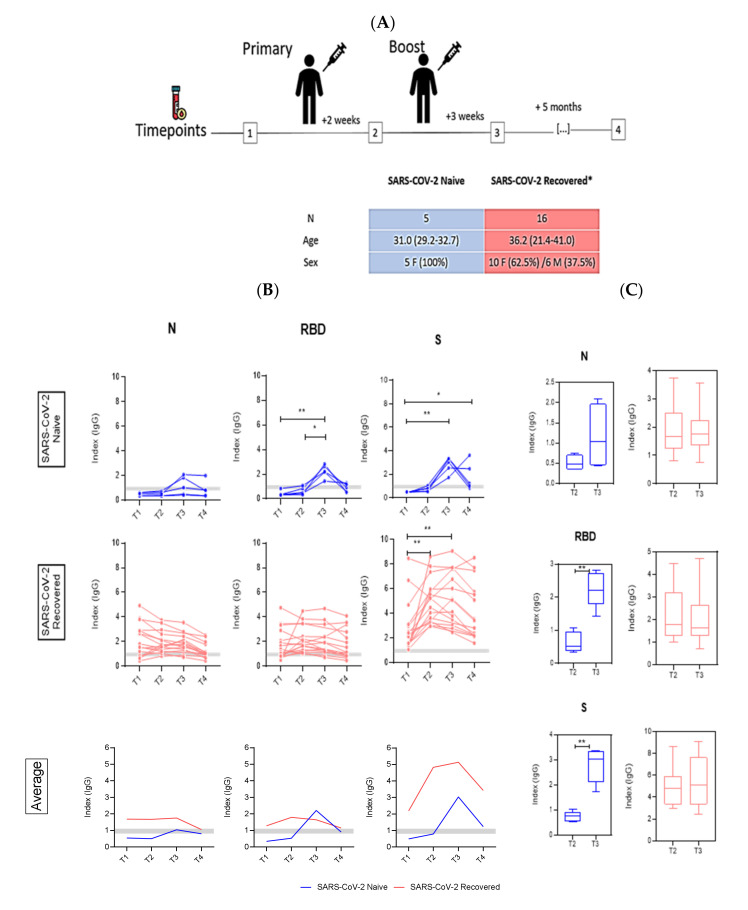
Antibody responses following CoronaVac vaccination in SARS-CoV-2-naïve (blue) and -recovered (red) individuals. (**A**) Study design showing the blood collection timepoints. (**B**) Kinetics of anti-nucleocapsid (N), anti-spike (S), and anti-RBD IgG antibodies in vaccinated (naïve, recovered, and average) individuals. The cut-off for samples to be considered positive was ≥1.1 and the borderline zone ranges from 0.8–1.1 (horizontal gray bars). Statistics were calculated using Kruskal–Wallis test. (**C**) Comparison between timepoints after first (T2) and second dose (T3) in naïve and recovered individuals using the unpaired Mann–Whitney test. * *p* < 0.05, ** *p* < 0.01.

We recruited twenty-one health care workers who received the two doses of the CoronaVac vaccine, sixteen of which had previous history of SARS-CoV-2 infection as confirmed by qPCR. The mean age of the five SARS-CoV2-naïve subjects was 31 years (29.2–32.7), and all were females. The SARS-CoV2-recovered group was composed of 10 females (62.5%) and 6 males (37.5%), with an average age of 36.2 years (21.4–41) (Figure 1A). 

All participants had blood collected at different timepoints before and after vaccination and tested by ELISA against three distinct SARS-CoV-2 antigens. At baseline, SARS-CoV2-naïve individuals had undetectable levels of antibodies to SARS-CoV-2 antigens (Figure 1B). After full vaccination, anti-nucleocapsid IgG antibodies were detectable in two out of five COVID-19-naïve individuals, whilst antibodies against RBD and S were detectable in all naïve subjects. The antibodies’ peak was observed after the second vaccine dose (T3), with a significant difference when compared to T1 (*p* < 0.01) and T2 (*p* < 0.05). After that, a decline in antibodies was observed (T4). Responses against N and RBD were not significantly different between the baseline (T1) and the last point (T4), but antibodies against S remained above index 2 for two individuals (the other two had borderline results). In contrast, most SARS-CoV2-recovered individuals had detectable antibodies for all three tested antigens at T1 although two individuals had undetectable anti-N responses, and four had undetectable anti-RBD antibodies. No significant differences in detection of anti-N and anti-RBD were observed at any timepoint. As for anti-S, there were significant differences between T1 and T2 (*p* < 0.01) and T2 to T3 (*p* < 0.01). When we compared the average ELISA’s index values for naïve and SARS-CoV2-recovered subjects, indexes of anti-N and -S were higher in those previously infected by SARS-CoV2. When considering the average humoral responses against RBD, we noticed a distinct peak after the second vaccine dose in naïve individuals, higher than the IgG levels in recovered subjects (Figure 1B, lower panel). Significant differences in antibody levels against RBD and S were observed between the two vaccine doses (T2 and T3) for naïve individuals. In contrast, SARS-CoV2-recovered subjects showed no additional increase in antibody levels against any of the three antigens following the second dose (*p* < 0.01) (Figure 1C).

**Figure 2:**
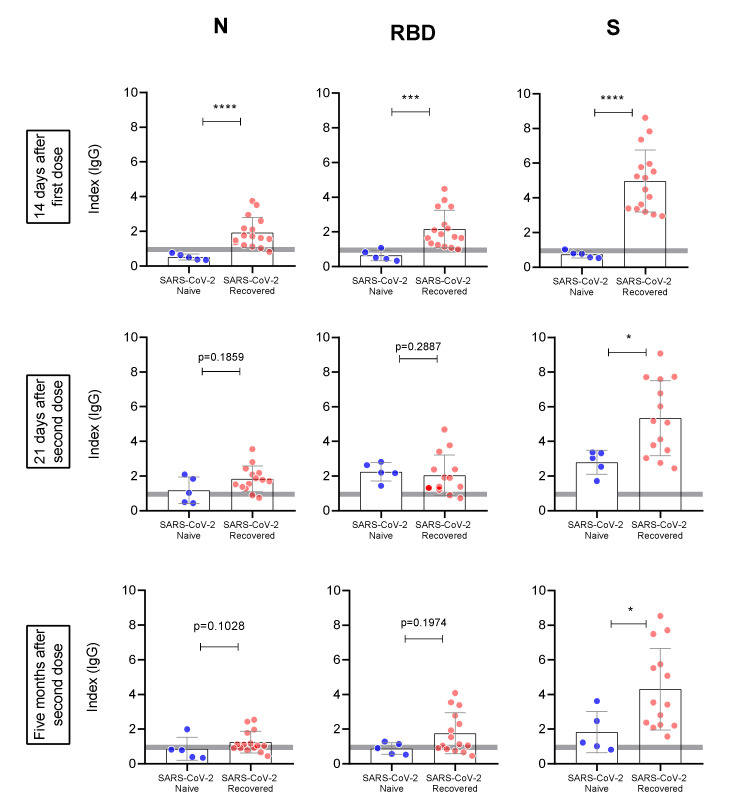
Evaluation of antibody response against N, RBD, and S between naïve (blue) and recovered (pink) individuals. The antibody response was evaluated in each timepoint after vaccination (T2, T3, and T4, respectively). Statistics were calculated using the unpaired Mann–Whitney test. Horizontal gray bars in A and B represent the cutoff and indetermined zone of the ELISA tests. * *p* < 0.05, *** *p* < 0.001, **** *p* < 0.0001.

In addition, when we compared the individual anti-N, anti-RBD, and anti-S responses between groups (Figure 2), significant differences between naïve and recovered individuals were observed after the first dose for all measured antibodies (*p* < 0.0001 for anti-N and anti-S and <0.001 for anti-RBD). Three weeks after the second vaccine dose, antibody levels against N and RBD became similar in SARS-CoV2-naïve individuals compared to those previously infected. Nonetheless, anti-S levels remained consistently higher in recovered subjects (*p* < 0.05). This same pattern was observed five months after the second dose.

**Figure 3:**
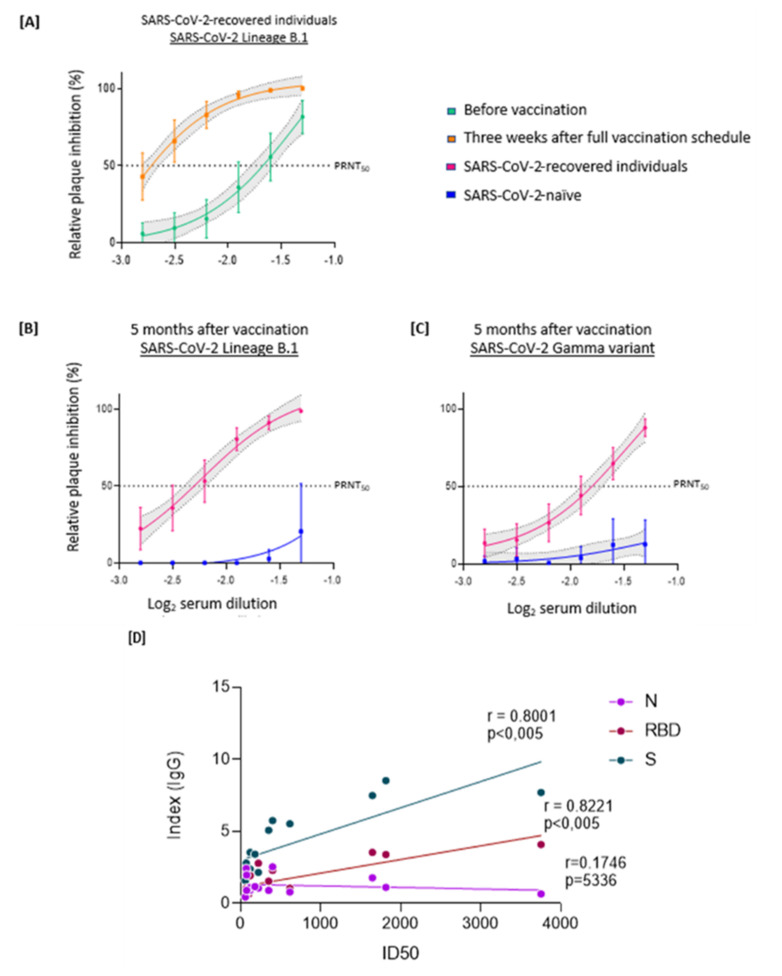
Virus-neutralizing antibodies against SARS-CoV-2 lineages B.1 and Gamma variant in SARS-CoV-2-naïve or previously infected vaccinated individuals. The PRNT50 represents the highest sample dilution that showed capacity to reduce by 50% the viral plaque formation based on control wells inoculated with SARS-CoV-2 without serum. Each point represents the mean of all plasma samples at each dilution, shown as log10; error bars represent 95% CI. (**A**) Sera from COVID19-recovered individuals were evaluated in two timepoints: before vaccination and three weeks after the second dose against lineage B.1 (underlined). Sera from either naïve or previously infected individuals were analyzed for their virus-neutralizing capacity, five months after full vaccination, against the virus B.1 lineage (underlined) (**B**) or Gamma variant (underlined) (**C**). (**D**) Correlation of PRNT 50 with IgG index against S glycoprotein (r2 = 0.6401), RBD (r2 = 0.6758), and N protein (r2 = 0.03051) (Spearman’s correlation, r; a linear regression was used to calculate the fit, r2).

To determine the production of neutralizing antibodies against SARS-CoV-2 and evaluate their longevity, we tested all sera by PRNT50 against a virus of the B.1 lineage as well as against the Gamma variant. We compared levels of neutralizing antibodies in SARS-CoV-2-recovered individuals before and three weeks after complete vaccination. Pre-vaccination sera had average PRNT50 values of near 1:40 and three weeks after the second dose titers were close to 1:640, there was a 16 times increase (*p* = 0.0009) (Figure 3A). In contrast, sera from SARS-CoV2-naïve individuals, analyzed five months after the second vaccine dose, had no detectable neutralizing activity against lineage B.1 or Gamma variant (Figure 3B,C). Again, SARS-CoV2-recovered subjects retained considerable neutralizing activity against SARS-CoV-2 5 months after vaccination, and the antibodies were able to neutralize both the B.1 lineage and the Gamma variant (Figure 3B,C). We evaluated the correlation between specific IgG levels and neutralizing antibodies 5 months after completion of the vaccine regimen. ELISA indexes obtained for the SARS-CoV-2-recovered individuals correlated with the observed IC50. A strong correlation (r > 0.8) was found for proteins S and RBD, whereas protein N showed no correlation (Figure 3D), as expected.

## 4. Discussion

As of January 2022, almost a third of the 9.8 billion COVID-19 vaccine doses delivered globally corresponded to the inactivated vaccine made by Sinovac. Most of the distributed doses were used in China, but a significant part of the vaccine production was sent to more than 110 countries, particularly the poorer ones [5]. Until May 2021, CoronaVac accounted for about 75% of the vaccine doses administered in Brazil although this proportion has declined as the Brazilian vaccination rollout progressed [15]. We evaluated and compared antibody responses triggered by the inactivated vaccine made by Sinovac in two distinct groups: SARS-CoV2-naïve and COVID-19-recovered individuals. Humoral responses were detected in individuals from both groups albeit subjects that had previous infections responded more intensely. A powerful boost effect was also observed in relation to neutralizing antibodies. Our data are consistent with other recent studies on mRNA vaccines [8,9,10,11], indicating robust boosting of antibody responses in SARS-CoV2-recovered subjects after vaccination. The observed strong correlation between ELISA IgG titers and PRNT50 was similar to other studies with SARS-CoV2-recovered individuals [16,17]. Despite the boost effect caused by the first dose in SARS-CoV2-recovered subjects, the second dose seems to add very little to the overall humoral responses. Our study population, which included only subjects that had mild infections with no need of hospitalization, corresponds to the majority of SARS-CoV-2 infections.

It is important to note, nonetheless, that although specific anti-SARS-CoV2 immunity is strongly boosted upon the second vaccine dose in individuals vaccinated with the inactivated COVID-19 vaccine and with no previous history of COVID-19, measurable immune responses tend to decrease sharply in the following five to six months as previously published [18]. Whether this correlated with lack of protection or not, however, remains to be determined.

Although important and consistent, our results were obtained from a small cohort of CoronaVac-vaccinated individuals, and that is an important limitation of the work. Additionally, our cohort included individuals who were not hospitalized upon SARS-CoV-2 infections, and it may be important to also evaluate patients who presented more severe manifestations. The time baseline (3.6 to 10.9 months after infection) is relatively large considering the small size of the study cohort, and that should also be noted. Given the relatively short timeframe of this study, future experiments are necessary to look into the longevity of humoral responses and investigate potential differences in long-term immunological memory. Cellular responses were not evaluated in this study.

Nonetheless, our data revealed distinct responses after CoronaVac vaccination based on prior SARS-CoV-2 exposure, suggesting that a single vaccine dose may be sufficient to induce high levels of anti-SARS-CoV2 antibodies—including neutralizing ones—in individuals with a history of previous infection with the virus. This could have important implications for vaccination strategies in scenarios where vaccine availability is a limitation factor for pandemic control.

## Data Availability

Not applicable.
